# Bones on fire: illuminating osteomyelitis through the radiant lens of ^18^F-FDG PET/CT

**DOI:** 10.3389/fimmu.2024.1378409

**Published:** 2024-03-12

**Authors:** Mei Yang, Quanhui Tan, Zhenghao Tang

**Affiliations:** Department of Infectious Diseases, Shanghai Sixth People’s Hospital Affiliated to Shanghai Jiao Tong University School of Medicine, Shanghai, China

**Keywords:** ^18^F-fluorodeoxyglucose (FDG), inflammation, infection, osteomyelitis, review

## Abstract

Osteomyelitis is an inflammatory process that is caused by an infecting microorganism and leads to progressive bone destruction and loss. Osteomyelitis can occur at any age and can involve any bone. The infection can be limited to a single portion of the bone or can involve several regions, such as marrow, cortex, periosteum, and the surrounding soft tissue. Early and accurate diagnosis plays a crucial role in reducing unnecessary treatment measures, improving the patient’s prognosis, and minimizing time and financial costs. In recent years, the use of functional metabolic imaging has become increasingly widespread. Among them, ^18^F-FDG PET/CT has emerged as a cutting-edge imaging modality that combines anatomical and functional metabolic information. It has seen rapid development in the field of infectious diseases. ^18^F-FDG PET/CT has been demonstrated to yield acceptable diagnostic accuracy in a number of infectious and inflammatory diseases. This review aims to provide information about the ^18^F-FDGPET/CT in the use of chronic osteomyelitis,osteomyelitis secondary to a contiguous focus of infection and osteomyelitis associated with peripheral vascular disease.

## 1 Introduction

Osteomyelitis is a condition characterized by inflammation caused by pathogenic bacteria attacking various components of the bone and its surrounding tissues, such as the periosteum, cortex, osteophytes, marrow, or adjacent structures. This inflammatory process typically results in bone destruction and loss (1). There are more than 12 different classifications of osteomyelitis, but the Waldvogel classification system is used in the clinical setting. Since the staging of the disease is usually directly related to the degree of tissue damage, it is necessary to distinguish between acute and chronic infections (2). Then, depending on the source of infection, osteomyelitis is subsequently categorized as hematogenous osteomyelitis, osteomyelitis secondary to successive foci of infection, and osteomyelitis associated with peripheral vascular disease. Of course, any type of osteomyelitis can develop from the acute phase and persist into the chronic phase of the disease.

The unique anatomy of the bone and its surrounding tissues contributes to the destructive nature of osteomyelitis. Inflammatory cells and other factors not only cause damage to bone tissue but also obstruct blood vessels, resulting in the formation of necrotic bone. Consequently, the presence of necrotic bone makes treatment challenging as it can serve as a refuge for the causative bacteria, rendering them difficult to eliminate even with antibiotics. Osteomyelitis can result in varying degrees of functional impairment, reduced quality of life, and in severe cases, disability and paralysis. Therefore, early diagnosis and timely intervention are crucial in managing osteomyelitis. The isolation of microorganisms from affected sites remains the “gold standard” for diagnosing osteomyelitis. However, imaging techniques are also necessary to locate the lesions (3), CT crosses the resolution of soft tissue and does not show bone marrow edema, and MRI, while reflecting early lesions with high sensitivity, is subject to metal interference (4), and all may not reflect the effectiveness of microbiological treatment as bone marrow edema may persist even after the infection has been controlled (5). Therefore, there is an urgent need for the development of new imaging tests to aid in the diagnosis of osteomyelitis.

^18^F-FDG is a radionuclide that shares a similar structure to glucose. It can enter cells through the glucose transporter (GLUT) located on the cell membrane (2). Inside the cell, it undergoes conversion to ^18^F-FDG-6-phosphate by the action of hexokinase. However, unlike glucose, 6-phosphate-^18^F-FDG is not further metabolized for energy and instead accumulates intracellularly. Therefore, the intracellular production of ^18^F-FDG-6-phosphate through internal irradiation reflects cellular hypermetabolism and its distribution (2).

In clinical practice, the concentration of ^18^F-FDG is typically quantified using standardized uptake value (SUV), which combines both anatomical and metabolic information. This quantification is achieved by combining ^18^F-FDG PET (positron emission tomography) imaging with computed tomography (CT) techniques. This hybrid approach allows for the visualization of both the anatomical structures and the metabolic activity of the tissues. By assessing the SUV, clinicians can gain insights into the degree of concentration of ^18^F-FDG and its uptake in different tissues or lesions, providing valuable information for diagnosis and treatment evaluation.

In 2001, the first PET/CT scanner was released, leading to rapid growth in the application of PET/CT in infectious diseases due to its ability to provide both metabolic and anatomical information (6). During tumor diagnosis, we have found that in addition to tumor cells that are highly metabolized and can be captured by PET, activated inflammatory cells such as neutrophils, monocytes/macrophages, and lymphocytes also express high levels of glucose transporters (especially GLUT1 and GLUT3) (7). Therefore, by detecting the accumulation of ^18^F-FDG in cells, we can obtain information about infection before any anatomical changes occur. Current guidelines have provided evidence-based indications for the use of ^18^F-FDG PET/CT in diseases such as sarcoidosis, peripheral bone osteomyelitis, suspected spinal infection, and fever of unknown origin (FUO). In this review, we summarize the use of ^18^F-FDG PET/CT in osteomyelitis, with a special focus on the common clinical conditions of chronic osteomyelitis, trauma-associated infections, periprosthetic infections, and osteomyelitis of the diabetic foot, and discuss the future directions of its application, which will provide a clinical reference for the use of ^18^F-FDG PET/CT in these conditions.

## 2 Applications of ^18^F-FDG PET/CT in osteomyelitis

### 2.1 Chronic osteomyelitis

Chronic osteomyelitis is a bone infection that typically arises from acute hematogenous seeding or penetrating injuries. It often occurs through contiguous spreading and persists for several weeks. During the chronic course of the disease, the periosteum thickens, muscles scar, and infected tissues combine with subcutaneous tissues, resulting in prolonged disease. The compromised blood supply in the affected area protects bacteria from antibiotics and the body’s immune response. CT and MRI are commonly used for diagnosing chronic osteomyelitis. However, their sensitivity and specificity in diagnosing the condition are average. A study by Ahmed Elsheikh et al. indicates that ^18^F-FDG PET/CT offers a non-invasive tool for diagnosing and localizing osteomyelitis, achieving a sensitivity of up to 94% and a specificity of 100%. The researchers compared clinical photos and ^18^F-FDG PET/CT images with clinical photos and plain X-ray images. Based on this comparison, consulting surgeons were able to identify the areas of infection and plan the extent of surgical debridement. The study found that ^18^F-FDG PET/CT can assist surgeons in differentiating an infected or necrotic bone from a healthy bone during surgery, surpassing the utility of plain X-ray imaging (2). Furthermore, Hulse et al. found that PET/CT demonstrated superiority over the emerging technology of PET/MRI in accurately delineating and localizing infections and demarcating bones (8). However, it should be noted that FDG PET/MRI is still an emerging technology and extensive prospective trials are required to establish its clinical value.

From the observations above, it is evident that ^18^F-FDG PET/CT not only aids in the diagnosis and localization of osteomyelitis lesions but also surpasses other imaging modalities in surgical planning and determining the scope of the procedure. Therefore, in cases of chronic osteomyelitis with sequestrum formation, ^18^F-FDG PET/CT has demonstrated superiority over MRI. This might be attributed to the pathogenesis of chronic osteomyelitis, which involves the formation of a sequestrum. Sequestra are areas of devitalized bone with reduced proton content, resulting in a lack of sufficient MRI signal. While ^18^F-FDG PET/CT is the most commonly used tracer in PET imaging, there is ongoing research into the development of novel radiotracers targeting specific molecular pathways involved in osteomyelitis. These targeted tracers could enhance the sensitivity and specificity of PET imaging by highlighting key pathological processes such as bacterial infection, inflammation, or angiogenesis. This could provide valuable information for early detection, accurate localization, and guidance of targeted therapies.

However, In the past decades, a limited number of studies have incorporated this technique in osteomyelitis as shown in **Table 1**, exclusion of technique dated and limited number of studies.

**Table 1:** Results for Sensitivity, specificity, and accuracy for the diagnosis of osteomyelitis with ^18^F-FDG PET-CT of the current study literature.

	Year	Content	cases	Modality	Sensitivity(%)	Specificity (%)	AUC of SUVs combine with qualitative assessments(%)	AUC of qualitative assessments(%)	diagnostic criteria ABC
Hartmann	2007	chronic osteomyelitis	33	visual analysis	94	87			A
Sandip Basu	2014	infection in hip knee prostheses	221	visual and semi-quantitative analysis	hip81.8 knee94.7	hip93.1 knee88.2		hip87.4 knee91.5	A
Vera Wenter	2015	chronic osteomyelitis	215	visual analysis	88	76			A,B
Kirsten E van Vliet	2018	infected non-union	30	semi-quantitative analysis	65	77		70	A,B
Justin V. C. Lemans	2018	FRI	135	visual and semi-quantitative analysis	89	80	89	84	A,B
Martina Sollini	2019	infected non-union	47	visual and semi-quantitative analysis	92	68	79	72	A,B

FDG is 2-[^18^F]-fluoro-2-deoxy-d-glucose; PET is Positron emission tomography; SUV is Standardized uptake values; CT is Computed tomography; A indicates histopathological findings and/or microbiological evaluation°B indicates clinical follow-up, C indicates other imaging including CT\ultrasound and so on.

FDG is 2-[^18^F]-fluoro-2-deoxy-d-glucose;PET is Positron emission tomography;SUV is Standardized uptake values;CT is Computed tomography;A indicates histopathological findings and/or microbiological evaluation, B indicates clinical follow-up, C indicates other imaging including CT\ultrasound and so on.

### 2.2 Osteomyelitis secondary to a contiguous focus of infection

#### 2.2.1 Fracture-related infection

With the aging population, the increasing prevalence of osteoporotic fractures, and surgical fracture repair procedures, the incidence of fracture-related infections (FRIs) is also rising and is expected to continue doing so (9). FRIs not only lead to increased morbidity but also higher healthcare costs and prolonged hospital stays. However, diagnosing FRIs poses a significant challenge. It was only in 2017 that clear diagnostic criteria were defined, and they were subsequently updated in 2020 (10). Current imaging tests have their limitations, and none of them can independently provide an accurate diagnosis (11).

The latest review of imaging modalities of fracture-related infection systematic found that the sensitivities and specificities of ^18^F-FDG PET/CT in diagnosing FRI ranges between 0.86–0.94 and 0.76–1.00, much higher than others but the gold standard-WBC scintigraphy (100% and 89-97%) of nuclear medicine examinations (12, 13). Zhang etc. retrieved 22 diagnostic studies and performed Bayesian network meta-analysis and ranking meta-analysis, indicating ^18^F-FDG PET/CT had satisfactory accuracy in identifying or excluding FRI. The pooled sensitivity of ^18^F-FDG PET/CT to diagnose FRI was 0.89 (95% CI 0.81–0.94), although the bone scintigraphy was 0.94 (95% CI 0.85–0.98), the specificity of bone scintigraphy was 0.34(95% CI 0.08–0.75), more less than 0.78(95% CI 0.72–0.84) of ^18^F-FDG PET/CT. The outcomes of network meta-analysis and ranking of diagnostic tests also found that ^18^F-FDG PET/CT (3.78; 95% CI 0.14–11.00) was the highest superiority index in diagnosing suspected FRI. Of course, WBC scintigraphy is also a good choice for its sensitivity and specificity were 0.86(95% CI 0.53–0.97) and 0.96 (95% CI 0.92–0.98) respectively (14). In a study by Justin V. C. Lemans et al., ^18^F-FDG PET/CT demonstrated a qualitative assessment diagnostic accuracy of 0.83 and an AUC of 0.84. When combined with SUVs (standardized uptake values), the AUC further increased to 0.89. It is important to note that this includes the influence of normal inflammation during the early postoperative period (15, 16).

According to the latest consensus, radiological and/or nuclear imaging signs such as non-union, implant loosening, bone lysis, sequestra, and periosteal bone formation are suggestive criteria for diagnosing FRIs. Magnetic resonance imaging (MRI), which is commonly used in other types of osteomyelitis, has limitations in distinguishing between sterile inflammation, aseptic inflammation, and reparative fibrovascular scar tissue. Additionally, artifacts from internal fixation materials further hinder its effectiveness. As mentioned earlier, WBC scintigraphy, although comparable to ^18^F-FDG PET/CT, has limitations such as long inspection times, and manipulation of leukocytes (13), it also can not identify physiological uptake in the bone marrow (17). Therefore, ^18^F-FDG PET/CT holds promise as a convenient and streamlined nuclear medicine test, working in conjunction with WBC scintigraphy as the gold standard nuclear imaging technique for diagnosing infections in fracture-related infections.

**Figure 1:**
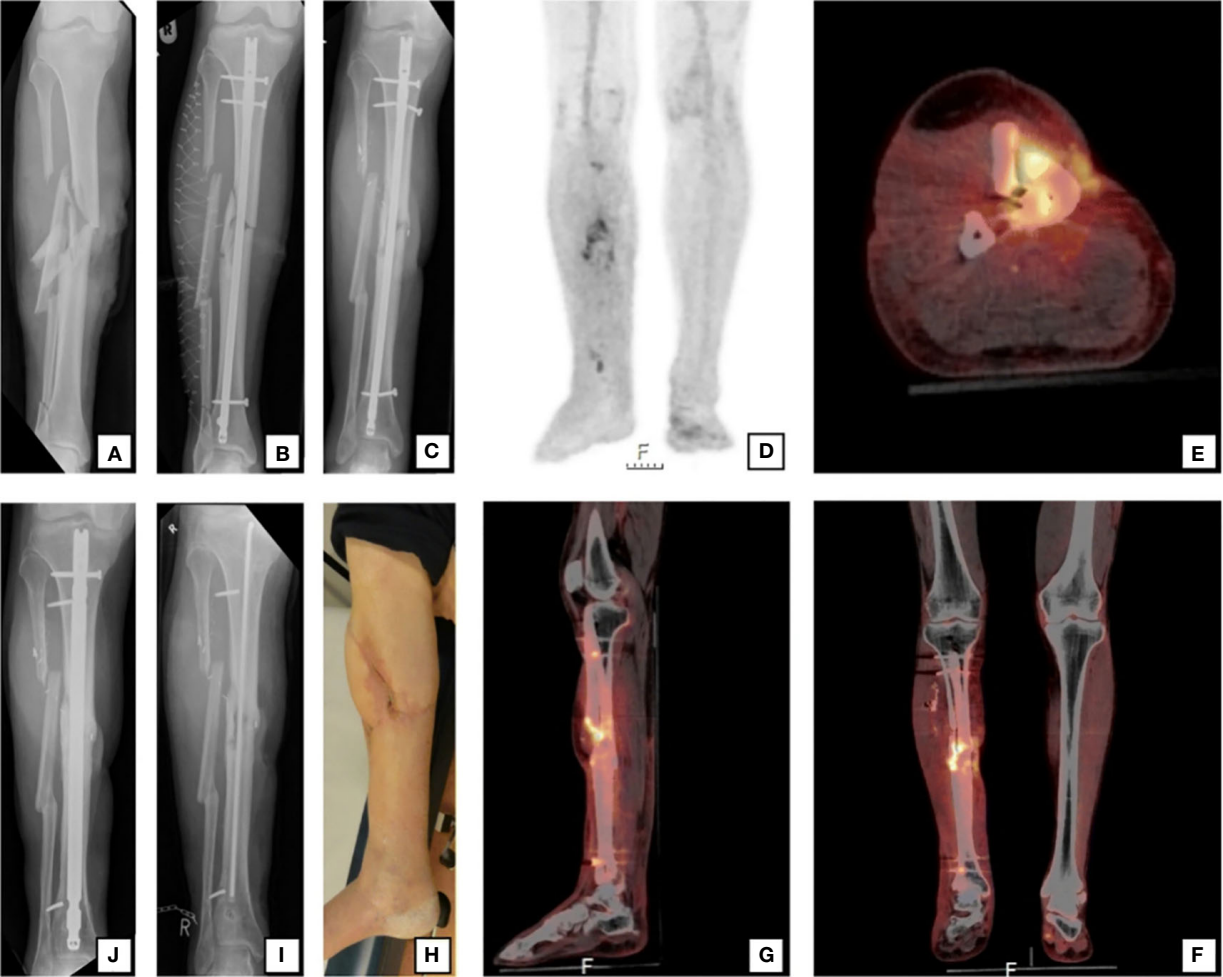
A 59-year-old man sustained a right-sided open crural fracture **(A)** which was treated with intramedullary nailing and a fasciotomy **(B)**. After several soft-tissue debridement procedures, the remaining soft tissue defect was eventually closed with a free musculocutaneous flap. After 20 months, there was a non-union with “autodynamization” of the intramedullary nail, demonstrated by broken interlocking screws **(C)**. The ^18^F-FDG PET/CT image **(D)** shows increased uptake around the fracture site in the tibial shaft and around the proximal and distal screws. The hybrid ^18^F-FDG PET/CT (**E** axial, **F** coronal, **G** sagittal) localize the suspected fracture-related infection (FRI) not only to the fracture site but also to the surrounding bone of the tibia around the fracture site which corresponds to the unstable scar overlapping the area of the non-union **(H)**. The intramedullary nail was removed, the tibia was reamed, the fracture site was debrided and an in-house, custom-made antibiotic nail was inserted **(I)**. FRI was confirmed by microbiological cultures and the patient was subsequently treated with antibiotics. One year after exchange nailing, fracture healing was successful **(J)** **Figure 1** reproduced with permission from Lemans, J.V.C., Hobbelink, M.G.G., IJpma, F.F.A. et al. The diagnostic accuracy of ^18^F-FDG PET/CT in diagnosing fracture-related infections. *Eur J Nucl Med Mol Imaging* 46, 999–1008 (2019). https://doi.org/10.1007/s00259-018-4218-6.

#### 2.2.2 Osteomyelitis associated with an infected prosthesis

With an aging population, joint replacements are on the rise every year and will continue to rise. Studies have shown that the average costs of one- and two-stage arthroplasty exchanges are 3.4 and 6 times higher than the cost of primary implantation (18). As a result, the cost of medical treatment due to PJI is also increasing every year (19). Most importantly, it is difficult to distinguish from a loose prosthesis in terms of clinical presentation, but the two are treated in very different ways. PJI progresses very rapidly and the longer it is delayed the more damage it does to the surrounding soft tissues some common PJI pathogens produce biofilms on the surface of the prosthesis that resists the use of antimicrobial drugs (20) and even can hide pathogens leading to increased false negatives in puncture specimens (21). A vicious circle is created which makes diagnosis more difficult. And the definition of PJI is also non-uniform, but neither the Musculoskeletal Infection Society (MSIS) nor the Infectious Diseases Society (IDSA) and the latest PJI scoring system considers imaging studies such as magnetic resonance imaging, computed tomography, and positron emission tomography should be confirmatory criteria (22–24). They do not apply to routine screening for PJI. However, they still are very important tools to assist clinicians in special situations, such as when surgical management is required.

At present, labeled leukocyte scintigraphy is considered the best nuclear imaging study for diagnosing PJI. But it is less accurate in the axial skeleton and the interpretation of images is very difficult and requires time-consuming and potentially dangerous *in vitro* labeling of autologous leukocytes. Of course, because of the lack of uniform interpretation criteria used, different FDG uptake patterns and interpretation criteria, and other defects, FDG PET is still no match for labeled leukocyte scintigraphy. However, studies have been conducted as early as 2003 indicated that ^18^F-FDG PET/CT scans alone showed the same sensitivity as combined bone scintigraphy (BS) and leucocyte scintigraphy (LS), although the specificity was slightly lower (25). In recent years, the development of technology and its joint use with CT scans greatly improved the diagnostic value of PJI.

In recent years, the combination of FDG PET with CT scans has significantly improved the diagnostic value of PJI. Scholars have proposed various interpretation criteria, including quantitative, semiquantitative, and qualitative visual assessment methods, often graded on a five-point scale. The study by Vera et al. indicated that the best image interpretation was obtained from a combination of visual interpretation and assessment of the FDG uptake pattern, as well as the judgment of the intensity of uptake in terms of SUVmax and SUVmax ratio, the accuracy of FDG was 76%. The specificity, PPV, NPV, and accuracy of PET/CT were higher than those of stand-alone PET (26).

Currently, ^18^F-FDG PET/CT is recommended for diagnosing lower-limb PJI involving knee and hip implants, while there is limited research on its application in upper limb joints, possibly due to the lower incidence of PJI in these joints. Importantly, using ^18^F-FDG PET/CT to assess PJI within one month of surgery is not advisable, as it can increase the likelihood of false test results (16).

**Figure 2:**
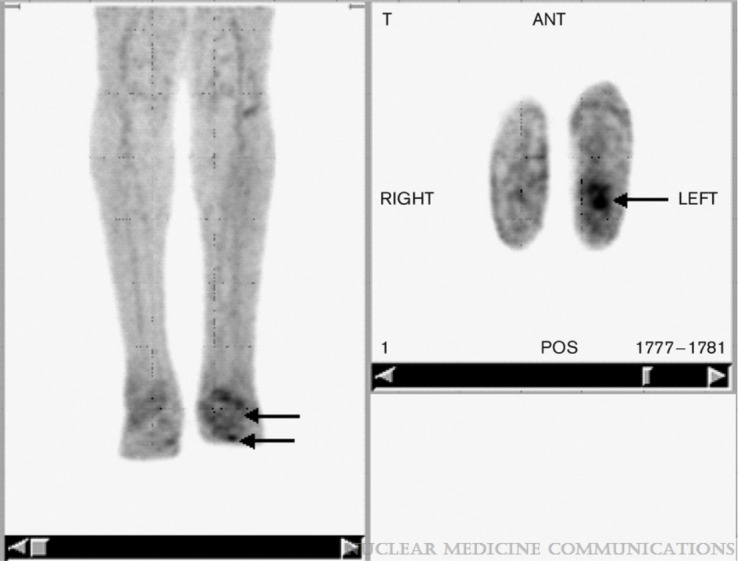
A 73-year-old male patient with pathological ^18^F-FDG PET/CT findings along the left hip prosthesis in contrast to the nonsuspicious prosthesis of the right hip. However, in this patient the intraoperative microbial culture was negative, and the PET scan was therefore rated false-positive. The FDG uptake was probably due to sterile inflammation occurring with implant loosening. **Figure 2** reproduced with permission from Wenter, V., Müller, JP., Albert, N.L. et al. The diagnostic value of [^18^F]FDG PET for the detection of chronic osteomyelitis and implant-associated infection. *Eur J Nucl Med Mol Imaging* 43, 749–761 (2016). https://doi.org/10.1007/s00259-015-3221-4.

### 2.3 Osteomyelitis associated with peripheral vascular disease

In more than two-thirds of cases, infection is the main cause of major lower limb amputation in diabetic patients with foot ulceration (27). The combination of poor blood flow, diminished pain perception, and compromised immune response in individuals with long-standing diabetes and peripheral vascular disease contributes to the development of difficult-to-heal infections that can spread to the surrounding soft tissues, potentially resulting in amputation or septic shock (28). Clinical guidelines recommend starting with X-ray as the initial imaging evaluation, followed by MRI (29). Additionally, peripheral insulin resistance and diabetic microangiopathy may lead to reduced FDG uptake at sites of inflammation. MRI has demonstrated superiority over ^18^F-FDG PET/CT or 99mTc-MOAB in detecting foot ulcer-associated osteomyelitis (30). When X-rays fail to provide a definitive characterization, MRI becomes the imaging modality of choice. However, in differentiating Charcot’s neuroarthropathy from osteomyelitis, FDG PET exhibits an overall sensitivity and accuracy of 100% and 93.8%, respectively, whereas MRI demonstrates 76.9% sensitivity and 75% accuracy (31), Therefore, X-rays are often negative during the early stages and primarily serve as a screening tool. It is important to note that MRI may yield false-positive results due to other diabetes-related complications such as neuroarthropathy and stress reactions, which can present with bone marrow and/or soft tissue edema similar to osteomyelitis (32). In this context, FDG PET imaging plays a crucial role in evaluating complicated and uncomplicated diabetic osteoarthropathy. MRI lacks the advantage of distinguishing between different pathological entities encountered in these patients. A meta-analysis of four clinical studies involving a total sample size of 178 patients with suspected diabetic foot osteomyelitis revealed that ^18^F-FDG PET/CT exhibited a specificity of 91% (95% CI: 85-96%), surpassing that of CT, MRI, bone imaging, and leukocyte imaging (32). Nonetheless, it is worth noting that existing diagnostic criteria lack uniformity, and the sample sizes of relevant studies have been limited. Thus, further large-scale multicenter studies utilizing bone biopsy as the gold standard are warranted to enhance our understanding in this area.

Studies have demonstrated that ^18^F-FDG PET/CT plays a valuable role not only in diagnosing osteomyelitis but also in monitoring the effectiveness of treatment. It has been observed that a reduction in SUVmax can be detected as early as two weeks into effective treatment, serving as an indicator of treatment response, even before changes in inflammatory markers become apparent (33). In contrast, changes in MRI images may take several months to manifest. Gannon J. Yu et al. proposed that activity confined to the margins of a destroyed or degenerated joint with bone-on-bone contact represents nonseptic inflammation, regardless of the intensity of uptake. And demonstrated by a prospective study with a sample size of 147 cases that ^18^F-FDG PET/CT achieved 100% and 100% sensitivity and specificity, respectively, in the initial diagnosis, 93% specificity in assessing response to treatment, and patients who discontinued antibiotics as a criterion were recurrent at 32 months of follow-up (34). However, it should be acknowledged that due to the atypical clinical presentation and rapid progression of certain cases of osteomyelitis, clinicians typically do not solely rely on MRI or inflammatory markers to discontinue antibiotic treatment or cease follow-up consultations. Therefore, further investigation is warranted to explore the clinical utility of ^18^F-FDG PET/CT in guiding treatment discontinuation decisions.

**Figure 3:**
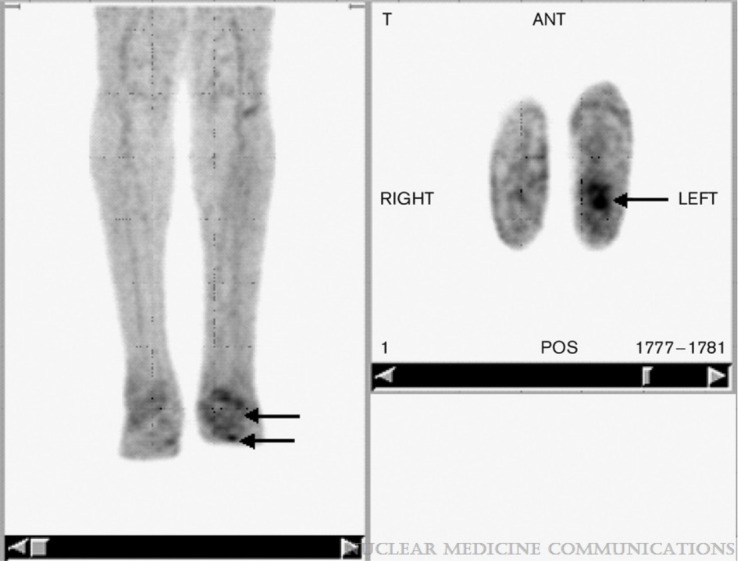
FDG PET in a patient with unilateral Charcot’s neuroarthropathy and foot ulcer. Note the focal uptake in the ulcer (arrows) in the transaxial images and the relatively low grade diffuse uptake in the neuropathic osteoarthropathy (arrows) clearly distinguishable from the uptake observed on the unaffected contralateral limb by visual inspection. **Figure 3** reproduced with permission from Basu, Sandip; Chryssikos, Timothy; Houseni, Mohamed; Scot Malay, D.; Shah, Jagruti; Zhuang, Hongming; Alavi, AbassNuclear Medicine Communications28(6):465-472, June 2007. doi: 10.1097/MNM.0b013e328174447f.

## 3 Summary and outlooks

^18^F-FDG PET/CT has demonstrated particular strengths in certain cases, including chronic osteomyelitis with sequestrum formation, suspected osteomyelitis in the axial skeleton, suspected peripheral diabetic foot infections or prosthetic joint infections that do not require immediate surgery or have a surgical history of more than six months with no orthopedic implants in place, and suspected dissemination of infectious foci, among others (35). As we can see the results for Sensitivity, specificity, and accuracy for the diagnosis of osteomyelitis with ^18^ F-FDG PET-CT of the current study literature in the **Table 1**.^18^F-FDG PET/CT has several limitations that should be considered. These include the high radiation dose associated with the procedure, the relatively high cost compared to other imaging modalities, the inability to identify specific pathogens for targeted antimicrobial therapy, and the need for patient preparation before the test, which can be more complex than a simple CT scan. Factors such as the post-operative time interval and the use of metabolic and immunosuppressive drugs, such as metformin, can also impact the accuracy of the results (36). It is anticipated that future technological advancements in ^18^F-FDG PET/CT will address some of these limitations. Accurate and early diagnosis of patients with insidious and atypical clinical presentations has always been a challenge in clinical practice. Combining multiple imaging modalities and incorporating other biochemical indicators is essential for achieving an accurate diagnosis. The goal is to reduce the time and costs associated with diagnosis and treatment by further developing complementary diagnostic techniques. Furthermore, the emergence of the novel nuclear medicine imaging instrument ^18^F-FDG PET/MRI has shown promising clinical value in neurological diseases (37). However, its application in osteomyelitis and comparative studies with ^18^F-FDG PET/CT still require further exploration.

As imaging technology continues to advance, there may be opportunities to integrate ^18^F-FDG PET/CT with other modalities such as MRI or ultrasound. Combining the anatomical detail of MRI with the functional information provided by PET imaging could enhance the accuracy of disease localization and characterization. This multimodal approach could facilitate more precise surgical planning, guidance of percutaneous interventions, and targeted tissue sampling. With the increasing availability of large datasets and advancements in artificial intelligence (AI) and machine learning (ML) algorithms, these technologies can be leveraged to improve the interpretation and analysis of ^18^F-FDG PET/CT images. AI and ML algorithms can aid in automating image interpretation, quantification, and pattern recognition. This could lead to improved diagnostic accuracy, efficiency, and standardization in the assessment of chronic osteomyelitis. In conclusion, the future of ^18^F-FDG PET/CT in chronic osteomyelitis holds promising developments in quantitative assessment, targeted imaging, theragnostic, multimodal integration, and AI-assisted analysis. These advancements have the potential to further enhance the role of ^18^F-FDG PET/CT in the diagnostic and therapeutic management of this complex bone infection.

## Author contributions

MY: Writing – original draft, Writing – review & editing. QT: Project administration, Supervision, Visualization, Conceptualization, Data curation, Formal analysis, Funding acquisition, Investigation, Methodology, Resources, Software, Validation, Writing – review & editing. ZT: Data curation, Methodology, Supervision, Conceptualization, Formal analysis, Funding acquisition, Investigation, Project administration, Resources, Software, Validation, Visualization, Writing – review & editing.
